# The impact of propranolol on nitric oxide and total antioxidant capacity in patients with resistant hypertension—evidence from the APPROPRIATE trial

**DOI:** 10.1186/s13104-020-05067-5

**Published:** 2020-04-21

**Authors:** H. N. Ranasinghe, N. Fernando, S. Handunnetti, P. N. Weeratunga, P. Katulanda, S. Rajapakse, P. Galappatthy, G. R. Constantine

**Affiliations:** 1 Department of Clinical Medicine, Faculty of Medicine, University of Colombo, Colombo, Sri Lanka; 2 Department of Pharmacology, Faculty of Medicine, University of Colombo, Colombo, Sri Lanka; 3 Institute of Biochemistry, Molecular Biology and Biotechnology, University of Colombo, Colombo, Sri Lanka

**Keywords:** Resistant hypertension, Oxidative stress, Antioxidant capacity, Nitrate, Nitrite

## Abstract

**Objectives:**

The objective was to assess the effect of propranolol on oxidative stress and anti-oxidant potential in patients with resistant hypertension as a secondary analysis of the APPROPRIATE trial. This randomized double blinded clinical trial recruited patients with resistant hypertension and allocated forty patients to propranolol and placebo in 1:1 ratio. The pro-oxidant state (nitrate and nitrite) was assessed using modified Griess assay. The total anti-oxidant capacity was measured using ABTS assay.

**Results:**

Analysis was performed for 18 patients from the propranolol group and 15 from the placebo group. A decline in end point ambulatory blood pressure (p = 0.031) and greater mean reduction in office SBP (29.7 ± 13.0 mmHg, p = 0.021) was noted in the propranolol arm. Nitrate and nitrite levels were lower at the end of a 90 day follow up period in both arms, with a greater mean reduction with propranolol. A significant increase in the AOC was noted in both arms with higher incremental value with Propranolol. The findings of this study do not demonstrate a statistically significant effect of propranolol on the oxidative stress/antioxidant balance in patients with resistant hypertension. The observed trends merit further evaluation.

## Introduction

Resistant hypertension (RH) is defined as uncontrolled blood pressure despite treatment (> 140/90 mmHg) with maximum tolerated doses of three antihypertensive agents of different classes used in combination. A meta-analysis published in 2018 estimated the prevalence of true-resistant hypertension at 10.3% (95% CI 7.6% to 13.2%) highlighting the burden and impact of the disease [1].

The role of pro-oxidant and antioxidant mechanisms in the pathogenesis of resistant hypertension remain unclear. Reactive oxygen species and reactive nitrogen species play an important role in the homeostasis of the vascular endothelium [2]. These molecules are implicated in endothelial dysfunction, vascular hyperreactivity, and vascular remodeling in patients with hypertension [3]. Mechanisms increasing oxidative stress such as reduction in superoxide dismutase and glutathione peroxidase activity, leading to elevated superoxide anions and hydrogen peroxide have been observed in newly diagnosed and untreated hypertensive subjects [4]. Reduced nitric oxide synthesis, decreased bioavailability of antioxidants and higher lipid hydroperoxide production have been demonstrated in experimental and human hypertension [5, 6]. Increased oxidative stress has also been demonstrated in patients with renovascular hypertension.

Propranolol is a nonselective beta blocker [7]. It is known to cause reduction in the oxidative stress markers free/protein malondialdehyde and lipid/protein hydroperoxides when used in patients with cirrhotic portal hypertension [8]. It also demonstrated protection against oxidative stress in iron overloaded rats [9]. The role of propranolol and its effects on oxidative stress in patients with resistant hypertension have not been evaluated in prior investigations.

This analysis focuses on the role of propranolol in reduction of oxidative stress in patients with RH included in the study. The physiological state of neutral oxidation is maintained by a delicate balance of pro-oxidants and anti-oxidants. It is hypothesized that in RH there is altered balance in this state with a shift towards a pro-oxidant state with low anti-oxidant capacity. The anti-oxidant defense mechanisms were evaluated by total anti-oxidant capacity.

## Main text

### Methods

The APPROPRIATE trial was a randomized, double-blind, placebo-controlled clinical trial to assess the efficacy of using propranolol in the management of patients with resistant hypertension (RH) at the medical outpatient clinics of the National Hospital of Sri Lanka. The inclusion and exclusion criteria for recruitment, screening, trial and experimental procedures have been published [10].

Participants fulfilling the inclusion criteria underwent a pre-specified screening procedure. During this screening procedure they were evaluated for compliance, lifestyle factors influencing blood pressure management and use of other medication triggering elevation of blood pressure. All patients subsequently underwent ambulatory blood pressure monitoring for exclusion of white coat hypertension. Only patients who fulfilled a diagnosis of hypertension following ABPM analysis were randomized for inclusion into the trial. Prior to randomization patients underwent clinical and laboratory evaluation for secondary hypertension. This included screening tests for renal artery stenosis, urinary metanephrines for Pheochromocytoma and aldosterone renin ratio for primary hyperaldosteronism.

#### Estimation of the nitric oxide activity by estimation of nitrates and nitrites

Serum NOx (NO_2_^−^ and NO_3_^−^) was used as a surrogate marker of NO activity. Blood samples collected were centrifuged, and separated sera were stored at − 20 °C till use. During analysis, the serum samples were thawed, then deproteinized by adding zinc sulphate. Ten microliters of 1.5 g/mL zinc sulphate solution was added to 1 mL of serum, vortexed for 1 min, and centrifuged at 10,000*g* for 10 min at room temperature (RT = 25 °C, i.e., the controlled temperature in the laboratory). The supernatant was pipetted out and centrifuged again at 10,000*g* for 10 min. The clear serum (100 μL) was applied in duplicate to a 96-well ELISA plate, 100 μL of vanadium (III) chloride (8 mg/mL) was added to each well (for reduction of nitrate to nitrite) followed by the addition of 100 μL of Griess reagent (equal mixture of 1% Sulphanilamide in 5% phosphoric acid and 0.1% N-(1-naphthyl) ethylenediamine hydrochloride in distilled water). The plates were incubated for 30 min at RT and the optical density measured at 540 nm using the microplate reader (Bio-Tek Instruments INC, USA). A two-fold dilution series (0.193–100 μM) of Sodium Nitrite (NaNO_2_) were prepared from 100 μM NaNO_2_ solution using distilled water. Each dilution (100 μL) was mixed with an equal volume of Griess reagent, and the optical density (OD) was measured at 540 nm. A standard curve was plotted against optical density and NaNO_2_ concentration.

Since serum nitrate is dependent on dietary factors, blood samples from all patients and healthy controls were collected in the morning between 0500 and 0600 h to minimize the dietary effect.

#### Estimation of serum total antioxidant capacity (AOC) with the ABTS decolourization method

Serum AOC levels were tested using 2,2′-azinobis-(3-ethylbenzothiazoline-6-sulfonic acid) (ABTS) decolorization method and expressed as serum Trolox equivalent antioxidant capacity (TEAC). Radical cations were generated by the oxidation of ABTS with potassium persulfate (K_2_S_2_O_8_). ABTS working solution was prepared by diluting the freshly prepared ABTS stock solution in 40-fold with 5 mM phosphate buffered saline (PBS). Distilled water and K_2_S_2_O_8_ in equal volumes (10 µL in each) were mixed with 800 µL of 5 mM PBS to prepare reagent blank. Test sera were mixed with ABTS working solution in 1: 9 ratio and kept exactly for 1 min to complete the scavenging process in dark. Samples were analyzed in duplicates and absorbance were measured at 734 nm against the reagent blank using spectrophotometer (UV spectrophotometer, Shimadsu, Japan) [7, 11]. A series of 6-Hydroxy-2, 5, 7, 8-tetramethyl-chroman-2-carboxylic acid (Trolox) two fold dilutions (400–12.5 µM) were mixed at the same ratio with ABTS working solution and the standard curve plotted using absorbance values. TEAC was calculated using the Trolox standard curve.

#### Statistical analysis

Statistical analysis was performed using SPSS version 16.0. Results were expressed as mean with SD for parametric values and median with interquartile range for non-parametric data. As the distributions for NOx, NO_2_ and AOC were non-parametric, appropriate statistical significance tests were used for comparison between groups and within groups. Within group data was analysed with the Wilcoxon signed rank test for related samples. Biochemical data for comparisons between the placebo and propranolol groups was performed using the Mann–Whitney U test. The null hypothesis was rejected at p < 0.05 for all analyses.

### Results

#### Baseline characteristics

A total of 160 patients were included based on initial randomization criteria to enter the screening phase. Following exclusion of white coat hypertension and secondary hypertension, forty patients with resistant hypertension were allocated into treatment and placebo at the prespecified allocation ratio. The two groups were uniform with respect to mean age, gender distribution, entry blood pressure and number of medications at baseline.

The final analysis for markers of oxidative stress and antioxidant capacity included 18 patients from the propranolol group and 15 patients from the placebo group of the clinical trial. Most patients in both propranolol group and the placebo group were females (72–73%). The mean age of participants in each group was 56 ± 9.9 and 56 ± 10.1 years respectively. Other baseline characteristics were similar across the groups. The baseline characteristics of these groups are shown in Table 1.

**Table 1:** Comparison baseline characters between the study group

Baseline characteristic	Propranolol	Placebo	P value
Mean age (years)	56.4 ± 9.9	56.9 ± 10.1	0.900
Weight (kg)	66.7 ± 10.7	71.8 ± 13.0	0.182
Height (cm)	152.9 ± 7.2	156.1 ± 9.1	0.223
BMI (kg/m^2^)	28.4 ± 3.8	29.3 ± 4.4	0.491
Gender (male:female)	5:13	4:11	0.546

#### Blood pressure measures at the end of intervention

The recorded office and ambulatory blood pressure measurements at the end of intervention are presented in the table given below. The trial was prematurely terminated due to achievement of all prespecified end points at the first interim analysis (Table 2).

**Table 2:** Office and ambulatory blood pressure measurements at the end of intervention

Blood Pressure	Propranolol (mmHg)	Placebo (mmHg)	P value
Office Blood pressure at end of intervention			
SBP (Systolic BP)	130.0 ± 13.2	139.93 ± 14.17	0.046
DBP (Diastolic BP)	77.8 (± 9.7)	82.66 ± 8.58	0.142
Mean reduction in office SBP	29.7 ± 13.0	18.07 ± 14.63	0.021
Mean reduction in office DBP	12.4 ± 12.8	6.33 ± 16.59	0.242
Mean ambulatory BP at the end of intervention			
SBP (Systolic BP)	137.3 ± 12.6	149.2 ± 12.5	0.080
DBP (Diastolic BP)	79.4 ± 5.5	84.1 ± 8.4	0.050
Mean reduction in ambulatory SBP	11.2 ± 14.7	4.1 ± 13.3	0.138
Mean reduction ambulatory DBP	5.4 ± 6.1	− 3.5 ± 15.6	0.031

#### Comparison of NOx and NO_2_^−^ levels (µM)

The baseline median with IQR in parenthesis, NO_x_ and nitrite (NO_2_^−^) levels in propranolol group (n = 18) 7.4 (4.7; 9.7) and 5.2 (0.5; 49.7) respectively. The median with IQR in parenthesis NO_x_ and (NO_2_^−^) after 90-day follow up were 5.0 (2.5; 6.2) and 0.8 (0.8; 1.2) respectively. The baseline median with IQR in parenthesis NO_x_ and (NO_2_^−^) levels in the placebo group (n = 15) were 5.8 (3.1; 8.4) and 1.6 (0.8; 6.4) respectively. The median with IQR in parenthesis NO_x_ and (NO_2_^–^) after 90-day follow up were 5.0 (3.0; 6.0) and 0.9 (0.8; 1.3) respectively. The reduction in NOx and NO_2_^−^ from baseline to endpoint was higher in the Propranolol group in comparison with the placebo group, however these observations were not statistically significant (p > 0.05). Comparative levels of NOx and NO_2_ at baseline as well as on follow up were not statistically significant in either group (Additional file 1: Table S1, Figures S1, S2).

#### Comparison of AOC levels in propranolol group (μM)

There was a notable increase in the median with IQR in parenthesis AOC levels in propranolol group 1705.7 (1467.3; 2914.2) after the 90 day follow up compared to baseline 337.5 (295.8; 369.8). This increase was also noted in the median with IQR in parenthesis placebo group 2133.6 (1563.9; 2912.6) after the 90 day follow up compared to respective baseline level 352.5 (294.0; 363.8). The individual increase in both groups from baseline was statistically significant (p = 0.000 for propranolol and p = 0.001 for placebo). The magnitude of increase in AOC was higher in the propranolol group, however these observations were not statistically significant (Additional file 1: Table S2, Figure S3).

### Discussion

Anti-oxidant capacity was significantly increased within groups. The absolute magnitude of increase was higher in the propranolol group. However, this did not reach statistical significance. Although low anti-oxidant capacity in hypertension is described in prior studies [12], no data available for resistant hypertension specifically. It is possible that increased AOC in this study may be result of reduction of blood pressure in both groups rather than a unique effect of propranolol. Expansion of the study to recruit a larger sample size and sub groups adjusted for magnitude of blood pressure decrease might be instrumental in uncoupling specific effects of propranolol on AOC.

It is interesting to note the decrease in NOx and NO_2_^−^ surrogate markers of NO at end point in both groups. However, as this decrease was not statistically significant in either group, no definitive conclusions were drawn. NO has complex roles in vascular endothelial function. In physiological states the molecule is largely protective, but induction of inducible nitric oxide synthase (iNOS) in disease states can lead to excessive NO reacting with superoxide, generates the oxidant anion peroxynitrite (ONOO^−^). This anion causes induces lipid peroxidation and nitrosation of amino acid residues, disrupting cell membranes, cell signaling and cell survival. Peroxynitrite also has proinflammatory effects [13]. Disease states such as hypertension lead to uncoupling of endothelial nitric oxide synthase (eNOS). The phenomenon of uncoupling refers to alteration of the functional state which is linked to increased monomerization of the enzyme. This process generates oxidatively active superoxides instead of NO. ROS and oxidative stress can also reduce the bioavailability of NO which decreases endothelium‐dependent vasodilation [14]. Therefore, trends in NO values should be interpreted with all the above determinants considered in future studies. Many dietary factors can also influence these values, and these were minimized with early morning, pre-breakfast blood samples.

## Limitations

The premature termination of the trial due to significant effect meant limitation of the sample size. The dietary influence was minimized by collecting samples in the early mornings prior to the intake of food. Evaluation of oxidative stress and antioxidant capacity requires a multi-pronged evaluation following this preliminary data and we propose future studies with measurement of all possible markers. This may include measurement of ROS and DNA/RNA damage, lipid peroxidation, and protein oxidation/nitration to represent the damage caused by oxidation as well as parallel evaluation of glutathione and AOC for anti-oxidant potential.

## Supplementary information

**Additional file 1: Table S1.** Comparison of NOx and NO_2_^−^ levels. **Figure S1.** Comparison of NOx concentration (µM) in plasma. **Figure S2.** Comparison of NO_2_^−^ concentration (µM) in plasma. **Table S2.** Comparison of Anti- Oxidant Capacity (AOC) levels between propranolol and placebo group. **Figure S3.** Comparison of AOC levels concentration (µM) in plasma.

## Abbreviations

NO: Nitric oxide
NOx: Total nitrites and nitrates
ELISA: Enzyme-linked immunosorbent assay
ROS: Reactive oxygen species
NHSL: National Hospital, Colombo, Sri Lanka
iNOS: Inducible nitric oxide synthase
eNOS: Endothelial nitric oxide synthase
AOC: Anti-oxidant capacity
RH: Resistant hypertension
SBP: Systolic blood pressure
DBP: Diastolic blood pressure
NaNO_2_: Sodium nitrite
IRQ: Interquartile range
