# Supplementary material for: The impact of propranolol on nitric oxide and total antioxidant capacity in patients with resistant hypertension—evidence from the APPROPRIATE trial
Source: BMC Res Notes. 2020 Apr 21;13:228. doi: 10.1186/s13104-020-05067-5 (PMC7175567; doi:10.1186/s13104-020-05067-5)

## Additional File 1: Tables

**Table S1: Comparison of NOx and $NO_2^-$ levels**

| **Intervention** | **Base-line Concentration** (μM) | | | | | | **Follow-up Concentration** (μM) | | | | | |
|---|---|---|---|---|---|---|---|---|---|---|---|---|
| **Propranolol** | **Mean** | **SD** | **Median** | **Q1** | **Q3** | **IQR** | **Mean** | **SD** | **Median** | **Q1** | **Q3** | **IQR** |
| Total $NO_x$ Levels | 9.1 | 10.0 | 7.4 | 4.7 | 9.4 | 4.7 | 4.9 | 2.1 | 5.0 | 2.5 | 6.2 | 3.7 |
| Total $NO_2^-$ Levels | 24.1 | 27.5 | 5.2 | 0.5 | 49.7 | 49.2 | 1.0 | 0.3 | 0.8 | 0.8 | 1.2 | 0.4 |
| | | | | | | | | | | | | |
| **Placebo** | **Mean** | **SD** | **Median** | **Q1** | **Q3** | **IQR** | **Mean** | **SD** | **Median** | **Q1** | **Q3** | **IQR** |
| Total $NO_x$ Levels | 5.6 | 2.5 | 5.8 | 3.1 | 8.4 | 5.3 | 4.9 | 3.1 | 5.0 | 3.0 | 6.0 | 3.0 |
| Total $NO_2^-$ Levels | 16.3 | 37.6 | 1.6 | 0.8 | 6.4 | 5.6 | 1.0 | 0.4 | 0.9 | 0.8 | 1.3 | 0.5 |

**Figure S1: Comparison of NOx concentration (μM) in plasma**

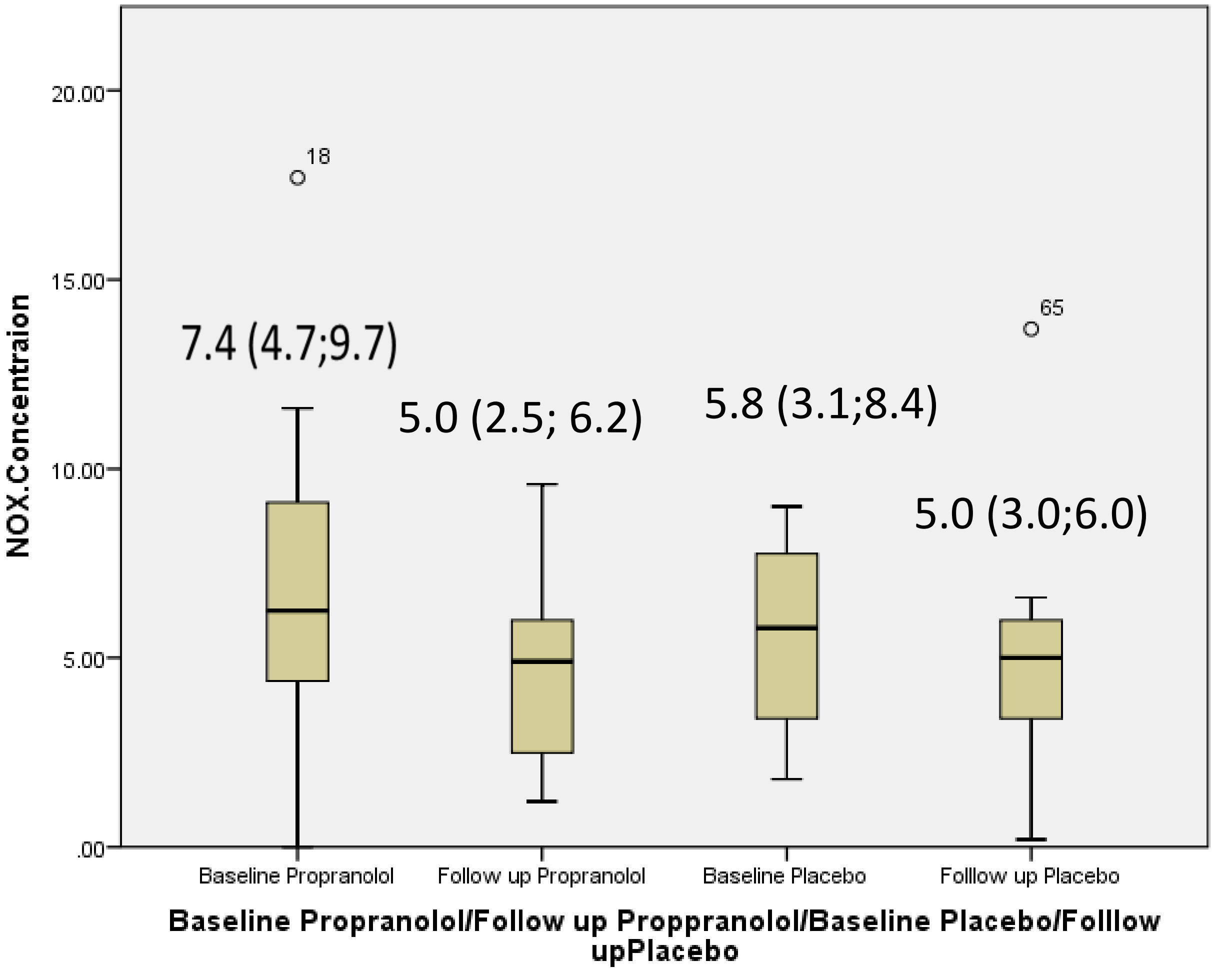

**Figure S2: Comparison of $NO_2^-$ concentration (μM) in plasma**

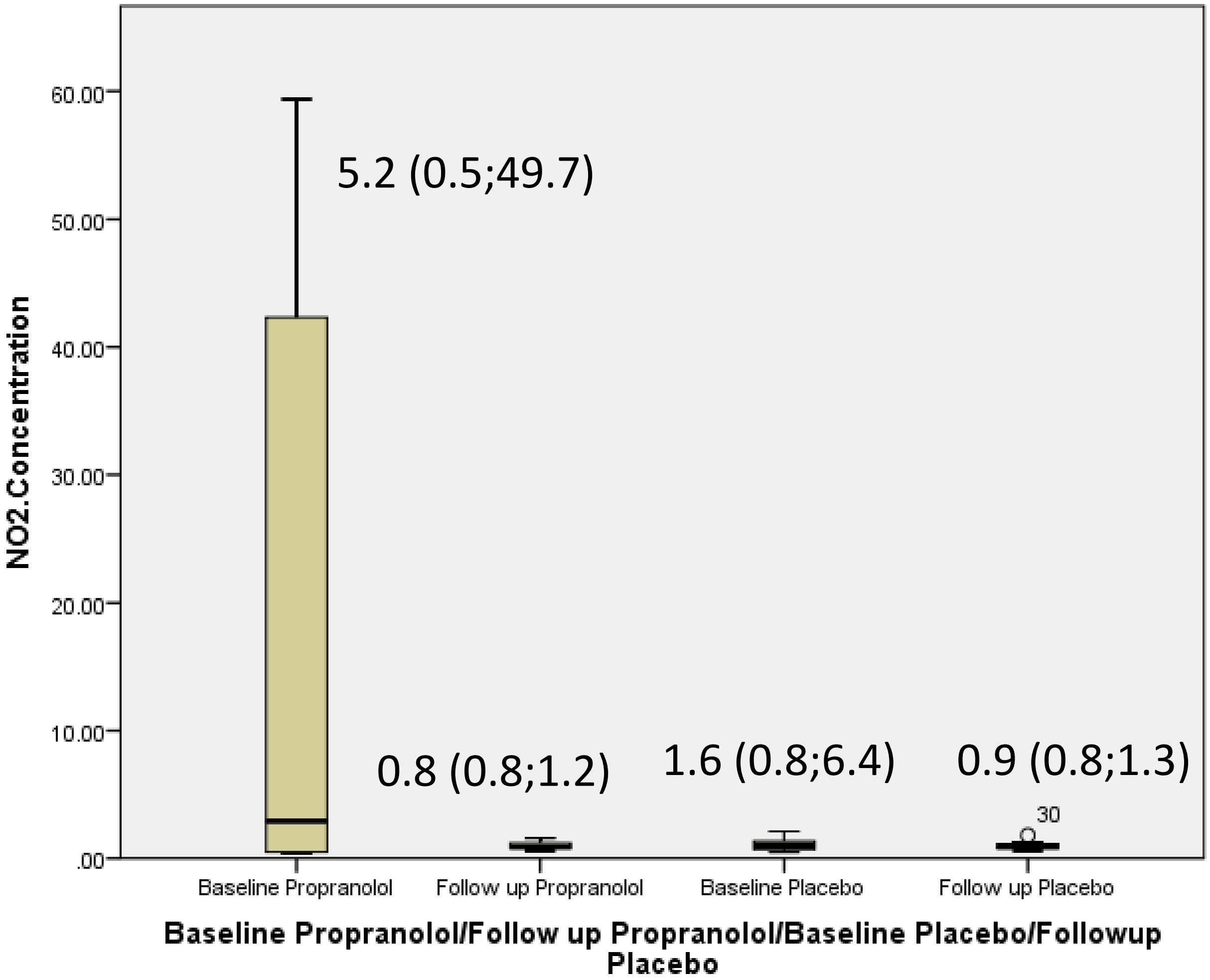

**Table S2: Comparison of Anti- Oxidant Capacity (AOC) levels between propranolol and placebo group**

| Intervention | Base-line Concentration (μM) | | | | | | Follow-up Concentration (μM) | | | | | |
|---|---|---|---|---|---|---|---|---|---|---|---|---|
| **Propranolol** | **Mean** | **SD** | **Median** | **Q1** | **Q3** | **IQR** | **Mean** | **SD** | **Median** | **Q1** | **Q3** | **IQR** |
| | 315.2 | 85.7 | 337.5 | 295.8 | 369.8 | 74.0 | 1924.2 | 1085.5 | 1705.7 | 1467.3 | 2914.2 | 1446.9 |
| | | | | | | | | | | | | |
| **Placebo** | **Mean** | **SD** | **Median** | **Q1** | **Q3** | **IRQ** | **Mean** | **SD** | **Median** | **Q1** | **Q3** | **IQR** |
| | 404.5 | 385.7 | 352.5 | 294.0 | 363.8 | 69.8 | 2195.1 | 961.9 | 2133.6 | 1563.9 | 2912.6 | 1348.7 |

**Figure S3: Comparison of AOC concentration (μM) in plasma**

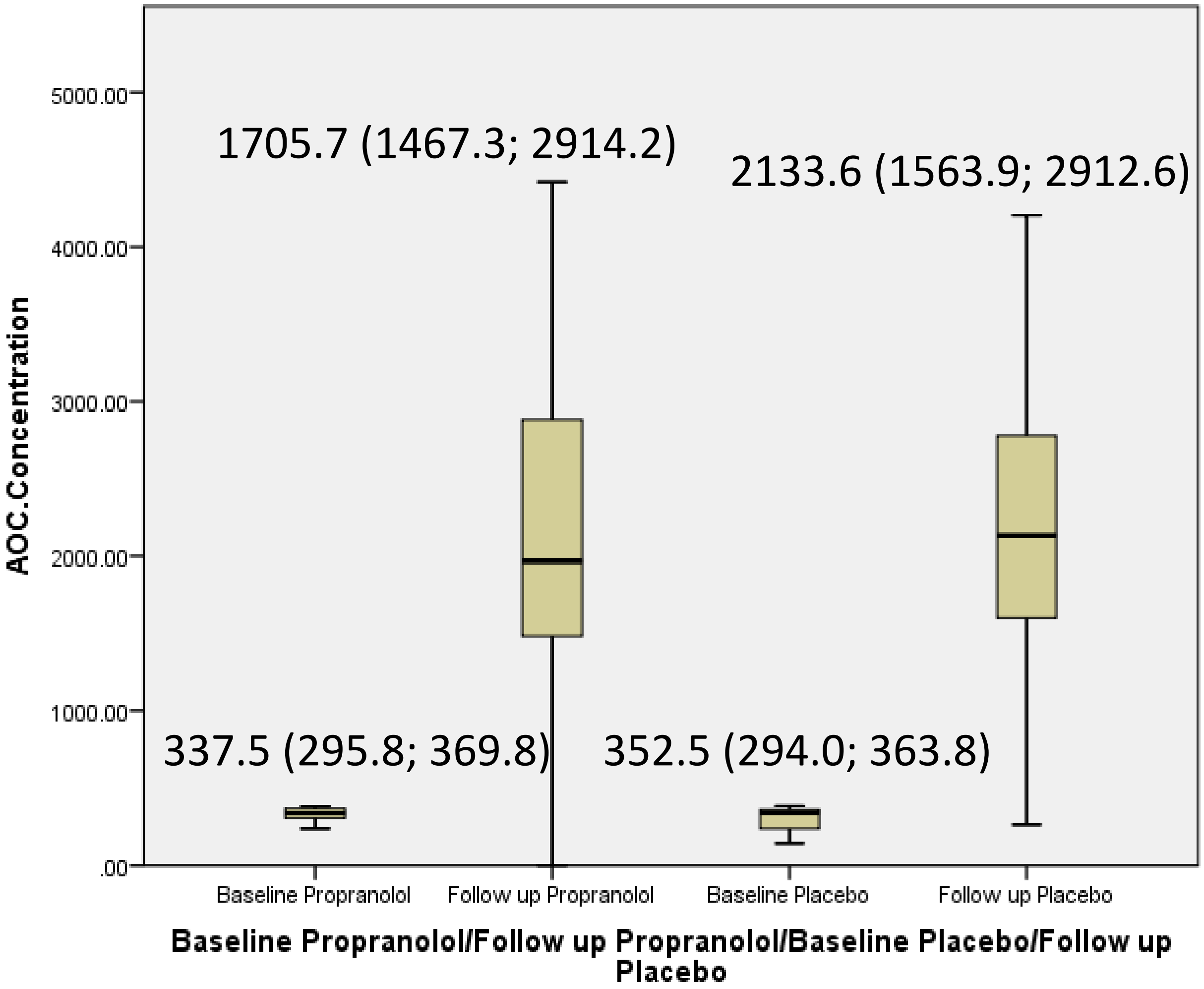

Supplement: Supplementary file 1 — Additional file 1: Table S1. Comparison of NOx and NO2− levels. Figure S1. Comparison of NOx concentration (µM) in plasma. Figure S2. Comparison of NO2− concentration (µM) in plasma. Table S2. Comparison of Anti- Oxidant Capacity (AOC) levels between propranolol and placebo group. Figure S3. Comparison of AOC levels concentration (µM) in plasma. [file 13104_2020_5067_MOESM1_ESM.pdf]
